# Levosimendan for Pediatric Anomalous Left Coronary Artery From the Pulmonary Artery Undergoing Repair: A Single-Center Experience

**DOI:** 10.3389/fped.2018.00225

**Published:** 2018-08-14

**Authors:** Chunrong Wang, Junsong Gong, Sheng Shi, Jianhui Wang, Yuchen Gao, Sudena Wang, Yong G. Peng, Jing Song, Yuefu Wang

**Affiliations:** ^1^Department of Anesthesiology, National Center for Cardiovascular Disease and Fuwai Hospital, Chinese Academy of Medical Sciences, Peking Union Medical College, Beijing, China; ^2^Department of Anesthesiology, UF Health Shands Hospital, University of Florida, Gainesville, FL, United States; ^3^Department of Anesthesiology, Harbor District Hospital of Zhengzhou First People's Hospital, Zhengzhou City, China

**Keywords:** levosimendan, pediatric anomalous origin of the left coronary artery from the pulmonary artery, coronary artery reimplantation, left ventricular dysfunction, postoperative outcomes

## Abstract

**Objectives:** Our aim was to retrospectively evaluate the benefit of levosimendan in certain complicated congenital heart procedures such as the pediatric anomalous origin of the left coronary artery from the pulmonary artery (ALCAPA) with moderate or severe cardiac dysfunction and its repair.

**Study Design:** We enrolled 40 pediatric patients with ALCAPA and moderate or severe left ventricular dysfunction. Patients who had a preoperative left ventricular ejection fraction (LVEF) of 50% or less and had undergone the surgical correction of their coronary artery through cardiopulmonary bypass met the criteria of our study. Twenty patients were given 0.1–0.2 μg/kg/min levosimendan at the induction of anesthesia, which lasted for 24 h. The remaining 20 patients were not given levosimendan.

**Results:** The mean preoperative LVEF in the levosimendan group was significantly lower than that in the non-levosimendan group (22.5 ± 10.7% vs. 31.8 ± 8.1%, *p* = 0.004). On postoperative day 7, the LVEF in the levosimendan group was still significantly lower (27.1 ± 8.9% vs. 37.5 ± 11.0%, *p* = 0.002). There was no significant difference in ΔLVEF detected on day 7 [median 30.8%, interquartile range (IQR) −4.4 to 63.5% vs. median 15.1%, IQR −3.5 to 40.0%, *p* = 0.560] or at follow-up of about 180 days (median 123.5%, IQR 56.1–222.6% vs. median 80.0%, IQR 36.4–131.3%, *p* = 0.064). There was no significant difference between the two groups in postoperative vasoactive-inotropic score (VIS) at any of the time points of 1, 6, 12, 24, and 48 h (*p* = 0.093). Three patients had to be supported by extracorporeal membrane oxygenation when difficulty appeared in weaning off cardiopulmonary bypass because of low cardiac output in the non-levosimendan group, but no patient needed extracorporeal membrane oxygenation after levosimendan infusion (*p* = 0.231). The length of intensive care unit stay (median 10.5 days, IQR 7.3–39.3 days vs. median 4.0 days, IQR 2.0–10.0 days, *p* = 0.002) and duration of mechanical ventilation (median 146.0 h, IQR 76.5–888.0 h vs. median 27.0 h, IQR 11.0–75.0 h, *p* = 0.002) were revealed to be longer in the levosimendan group. Peritoneal dialysis occurred in eight patients (40%) in the levosimendan group and two patients (10%) in the non-levosimendan group (*p* = 0.028). No significant difference was revealed in all-cause mortality within 180 days, which occurred in two patients (10%) in the levosimendan group and one (5%) in the non-levosimendan group (*p* = 1.00).

**Conclusion:** Levosimendan's unique pharmacological properties have strong potential for cardiac function recovery among pediatric patients with ALCAPA with impaired left ventricular function who have undergone surgical repair.However, any improvement from levosimendan on postoperative outcomes or mortality was not substantiated by this study and must be investigated further.

## Introduction

Anomalous origin of the left coronary artery from the pulmonary artery (ALCAPA), also known as Bland-White-Garland syndrome, is well-recognized as an uncommon congenital heart disease (1). It makes up a proportion of about 0.25–0.5% of all congenital heart diseases, and its incidence is about 1 per 300,000 newborns (1, 2). A high mortality rate (up to 90%) is reported if the defected is not repaired in the first postnatal year. ALCAPA is mainly characterized by chronic myocardial ischemia, even infarction, and various degrees of mitral regurgitation secondary to annual dilation or papillary muscle dysfunction. If the myocardium suffers steady long-term hypoperfusion, gradually, both subendocardial ischemia and fibrosis occurs, leading to arrhythmias and even sudden death (3, 4). In addition, either primarily compromised heart function or a cardiopulmonary bypass (CPB) procedure can result in postoperative low cardiac output syndrome (LCOS). This syndrome remains the most common complication of ALCAPA repair and occurs in as high as 25% of all cases (5). It has been suggested that preoperative systolic ventricular dysfunction could prolong CPB time or even lead to difficulty weaning from CPB. It can also become a risk factor for perioperative mortality (2). Thus, the appropriate selection of inotropes is a crucial strategy for procedures to repair ALCAPA, especially when the left ventricular ejection fraction (LVEF) is 50% or less.

The primarily perioperative objective is to maintain stable hemodynamics and avoid further cardiac function deterioration. However, there are limited perioperative management tactics for these objectives.

Levosimendan, a calcium-sensitizing inotropic agent, is a preferred inotrope that is able to enhance cardiac contractility without increasing cardiac oxygenation consumption. It has two principal mechanisms: (1) it enhances the sensitivity of cardiac troponin C to calcium and (2) it mediates the gateway of adenosine triphosphate-sensitive potassium, which is located at the vascular smooth muscle and mitochondrial inner membrane. Levosimendan performs these roles by improving contractility through positive inotropic function and dilating peripheral vessels and the coronary artery; it also has anti-ischemia effects (6–8). It has been widely used to treat heart failure in Europe (9), and studies have reported its increasing use among adult patients for surgery management (10, 11). In general, prophylactic levosimendan seems to be a promising intervention for improving cardiovascular function and it is an effective therapeutic approach for preventing perioperative LCOS in a surgical setting.

Despite a number of prospective or retrospective trials have been carried out on the practical applications of levosimendan in congenital cardiac surgery, proving that it can be well-tolerated, there is still no relevant report on ALCAPA repair. This retrospective analysis from our medical center is intended to explore the effect of levosimendan on the improvement of left ventricular function as well as in-hospital outcomes among pediatric patients with ALCAPA and LVEF of 50% or less undergoing coronary reimplantation.

## Materials and methods

### Patients and data collection

**Figure 1:**
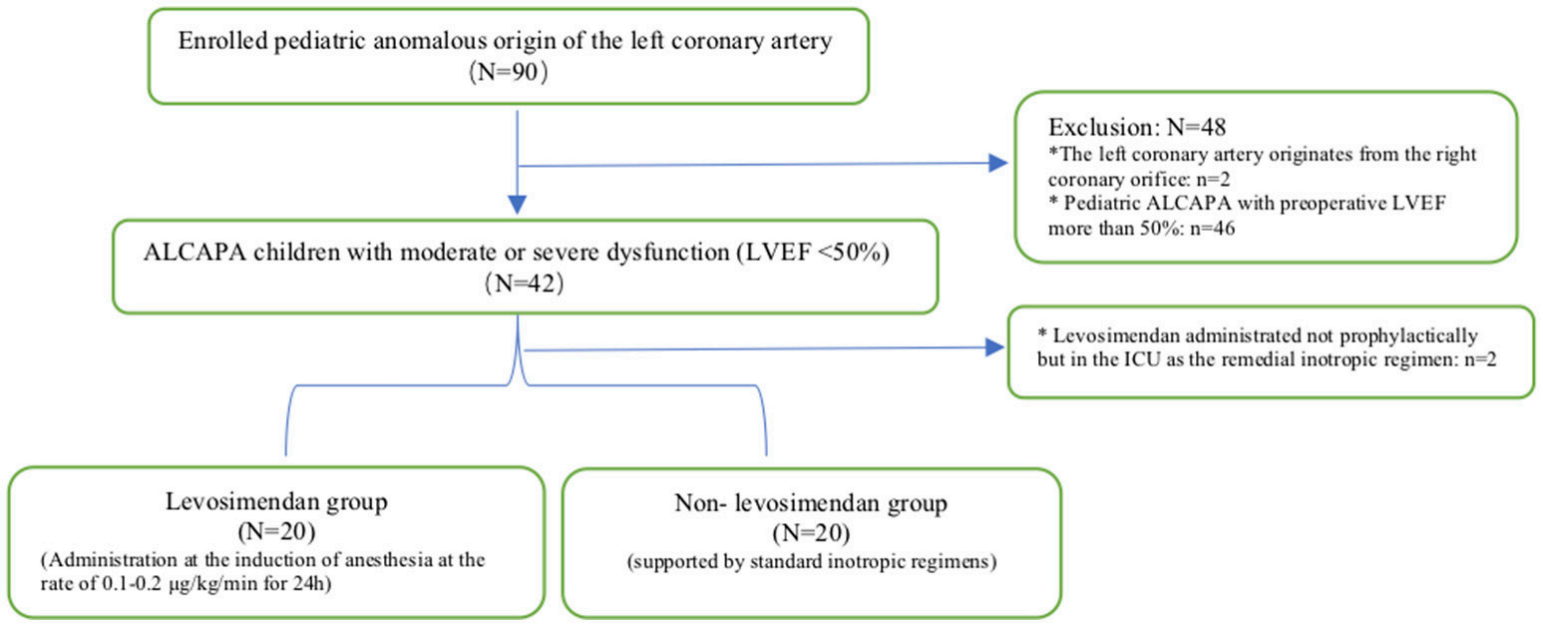
Enrollment procedure. ALCAPA, anomalous origin of the left coronary artery from the pulmonary artery; LVEF, left ventricular ejection fraction; ICU, intensive care unit. ^*^Represented the patient exclusion criteria.

The study protocol was approved by our hospital institutional review board. Figure 1 illustrates the enrollment process of the eligible pediatric ALCAPA population in our study scheme. Between 2010 and 2017, only 40 pediatric patients with ALCAPA in our medical center were admitted with moderate or severe left ventricular dysfunction, namely, with a LVEF of 50% or less. In addition, two patients treated with levosimendan as a remedial solution at the time of intensive care unit (ICU) admission were excluded from study. All patients received left coronary artery reimplantation, and 15 patients (37.5%) underwent concomitant mitral annuloplasty. Data were extracted retrospectively from our digital medical records, which covered the whole course from their initial hospitalization through the last follow-up visit.

### Definition of ΔLVEF

All of the patient's cardiac systolic functions were assessed by transthoracic echocardiography at three time points (preoperative, day 7, and at follow-up ~6 months later), and the baseline LVEF was collected on the day before surgery. The LVEF values on postoperative day 1 were not included because of incompleteness: there were missing data of more than 30%. We assumed the definition of ΔLVEF to evaluate cardiac function improvement, which was calculated as follows: (postoperative LVEF – preoperative LVEF)/preoperative LVEF × 100. After repair, baseline LVEF rose from 10 to 40%, which is different from a rise of 30–60%, and an ΔLVEF of 300 and 100%, respectively. The former would manifest a much more better recovery under the conditions of deteriorating cardiac function, which was the case before surgery, although both of the LVEF changes were 30%.

### Vasoactive-inotropic score (VIS)

The VIS has been proposed as another indirect measurement to assess the hemodynamic conditions, whether improved or deteriorated. In the study, VIS was applied as: dopamine (μg/kg/min) + dobutamine (μg/kg/min) + [100 × epinephrine (μg/kg/min)] + [100 × norepinephrine (μg/kg/min)] + [10,000 × vasopressin (U/kg/min)] (12). The VIS at 1, 6, 12, 24, and 48 h after surgical repair was calculated separately for each group.

### Criterion for acute kidney injury (AKI)

The pediatric-modified Risk, Injury, Failure and Loss, and End-Stage (pRIFLE) system has been validated as the most sensitive criteria for identifying acute kidney injury **(**AKI) (13). Additionally, the estimated glomerular filtration rate required for pRIFLE assessment was derived from the most unbiased and sensitive Schwartz model formula, which was based on postoperative serum creatinine levels and patient height (14). The AKI in our study was qualified as early as within 12 postoperative hours.

### Anesthesia protocol

The general anesthesia technique was based on individual anesthesiologist preference. During induction, appropriate dosages of intravenous agents such as ketamine, midazolam, rocuronium, and sufentanil were combined with facemask oxygen and oral intubation was carried out. Both invasive blood pressure monitoring and blood gas analysis were achieved by radial artery catheterization, and the right internal jugular vein was cannulated for central venous pressure or left atrium pressure monitoring. Throughout the procedure, anesthesia maintenance was achieved by the continuous infusion of a combination of dexmedetomidine, propofol, sufentanil, and rocuronium at the appropriate dosage; additionally, all patients were mechanically ventilated with mixed air and oxygen; the oxygen level was 40–45%.

### Surgical technique

The surgical approach was achieved via median sternotomy, CPB, and coronary reimplantation, but the simultaneous mitral valvuloplasty was only considered whenever necessary. CPB consisted of aorta and bicaval cannulation, blood priming, antegrade cardioplegic arrest, and then mild hypothermia. First, the pulmonary and aortic trunks were transected and then the left coronary artery and it's attached pulmonary wall were resected. Then the left coronary artery was re-implanted into the aorta and, whenever necessary, the resected pulmonary wall was made into a coronary tunnel if there was a long distance between the aorta and coronary ostia. The defect in the pulmonary trunk was repaired with a pericardial patch, and the anastomosis of each main artery was completed separately. According to the identification of the grade and severity of mitral regurgitation, mitral valvuloplasty was performed if needed.

### Administration of levosimendan

All intra-operative inotropic management strategies were at the discretion of the attending anesthesiologists. We divided the pediatric patients with ALCAPA into the levosimendan group (*n* = 20) and the non-levosimendan group (*n* = 20). For the levosimendan group, no initial loading dosage was administered, but they were given a 24-h, 0.1–0.2 μg/kg/min continuous infusion after the procedure. Both groups received 3 μg/kg/min dopamine as a routine inotrope when being warmed during CPB, and if necessary, other medical regimens or mechanical supports could be applied at any time as a rescue to maintain both sufficient cardiac contractility and stable hemodynamics.

### Endpoint

The primary outcome for our retrospective study was whether the prophylactic use of levosimendan could improve the cardiac function or not on day 7 and at follow-up to 180 days. Other outcomes included various in-hospital endpoints such as VIS, the length of ICU stay, duration of mechanical ventilation, peritoneal dialysis, and all-cause mortality though day 180. In addition, we analyzed the incidence of AKI, arrhythmia, the number of patients requiring perioperative circular support (as well as after being admitted to the ICU), and the rates of respiratory events such as re-intubation, tracheotomy, and postoperative pneumonia.

### Statistical analysis

We performed this retrospective analysis using SPSS, version 23 (IBM, Armonk, NY, USA).

Normally distributed continuous variables were presented as the mean ± standard distribution, whereas non-normally distributed data were presented as the medians with their interquartile ranges (IQR). Categorical variables were presented as frequencies and percentages. Group comparisons of the two types of variables above were used for the two-tailed *t*-test or the Mann–Whitney *U* test, chi-square test, or Fisher's exact test. Repeated measures analysis of variance was done when comparing the time course of the VIS between groups. We considered *p* < 0.05 as statistically significant.

## Results

### Study patient characteristics

Forty pediatric patients (21 males and 19 females, 2 months−12 years old) undergoing ALCAPA repair in our medical center were reviewed. 20 patients were given levosimendan at anesthesia induction and the other 20 were not. The median age of the whole population at repair was 7.5 months (IQR, 4.5–18.0 months). The median weight was 8.1 kg (IQR, 5.9–9.5 kg). The mean LVEF was 27.2 ± 10.5%, and the patients in the levosimendan group suffered more severe left ventricular dysfunction. There were eight patients who underwent concomitant mitral annuloplasty in the levosimendan group compared with seven in the non-levosimendan group. There was no significant difference between groups in demographic parameters or surgical data, including CPB time or aortic cross-clasp time (Table 1).

**Table 1:** Comparison of demographic parameters and surgical data.

	**Levosimendan (*n* = 20)**	**Non-levosimendan (*n* = 20)**	***p*-value**
Male, *n* (%)	9 (45%)	12 (60%)	0.749
Age at operation, months, mean ± SD	7.5 (3.0–13.5)	8.5 (6.0–21.3)	0.532
Height, cm, median (IQR)	68.5 (60.0–74.8)	73.5 (66.3–89.3)	0.088
Weight, kg, median (IQR)	6.5 (5.4–8.9)	8.5 (6.6–10.8)	0.058
Left ventricular aneurysm, *n* (%)	1 (5%)	4 (20%)	0.342
Concomitant mitral annuloplasty, *n* (%)	8 (40%)	7 (35%)	0.744
LVEF			<0.01
<20%, *n* (%)	13 (75%)	2 (10%)	
20–30%, *n* (%)	3 (15%)	7 (35%)	
30–50%, *n* (%)	4 (20%)	11 (55%)	
CPB, min, median (IQR)	102.0 (88.3–126.8)	105.5 (94.5–116.5)	0.850
Aortic cross-clasp, min, median (IQR)	60.5 (48.0–71.0)	67.0 (53.5–89.3)	0.176

*LVEF, left ventricular ejection fraction; CPB, cardiopulmonary bypass*.

### Primary outcome of cardiac function

**Figure 2:**
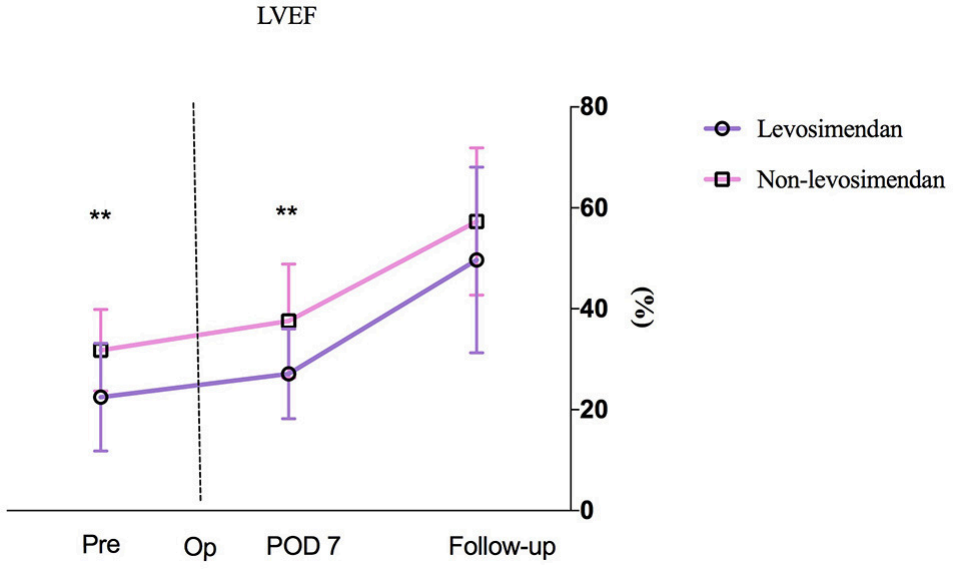
A patient's LVEF at three time points: preoperation, on day 7 after surgery, and at follow-up. No mean LVEF on day 1 between the levosimendan and the non-levosimendan groups was compared because there were missing data of more than 30%. LVEF, left ventricular ejection fraction; Pre, preoperation; Op, operation; POD, postoperation. ^**^p < 0.01.

**Figure 3:**
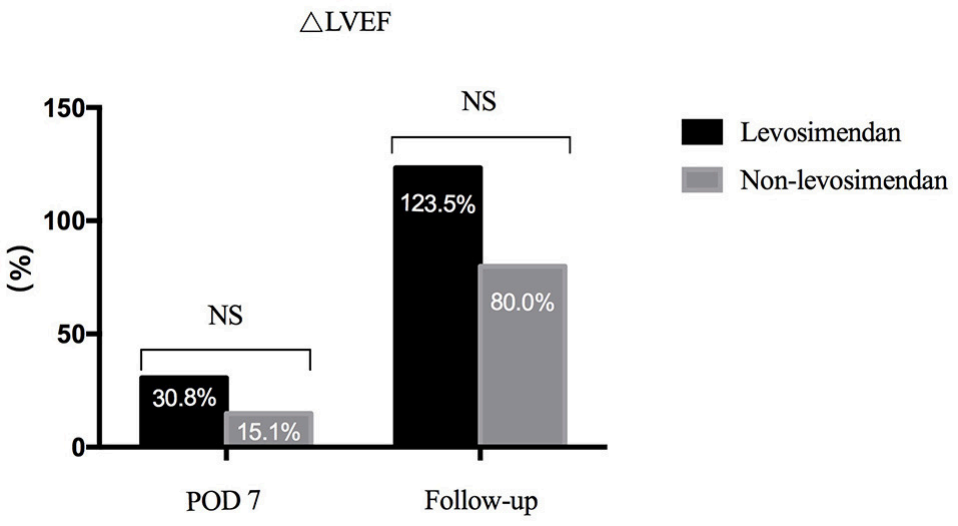
Bar graph showing the ΔLVEF of the two groups. On day 7, there was no significance between the levosimendan and the non-levosimendan groups (median 30.8%, IQR −4.4 to 63.5% vs. median 15.1%, IQR −3.5 to 40.0%, respectively). In addition, no significant difference was revealed at their follow-up (median 123.5%, IQR 56.1–226.0% vs. median 80.0%, IQR 36.4–131.3%). LVEF, left ventricular ejection fraction; NS, no significance; POD, postoperation.

Preoperative LVEF was revealed to be significantly lower in the levosimendan group than in the non-levosimendan group (22.5 ± 10.7 vs. 31.8 ± 8.1%, *p* = 0.004). Compared with the non-levosimendan group, on day 7 after coronary reimplantation, each median LVEF was still significantly lower in the levosimendan group (27.1 ± 8.9 vs. 37.5 ± 11.0%, *p* = 0.002; Figure 2). No significant difference in ΔLVEF was detected on day 7 or at follow-up ~180 days later (median 30.8%, IQR −4.4 to 63.5% vs. median 15.1%, IQR −3.5 to 40.0%, *p* = 0.560; median 123.5%, IQR 56.1–226.0% vs. median 80.0% IQR 36.4–131.3%, *p* = 0.064; Figure 3). In addition, at follow-up, LVEF in nine patients (50.0%) in the levosimendan group and in 14 patients (73.7%) in the non-levosimendan group had increased more than 50% with the exception of three patients who died in the ICU.

### VIS variance

**Figure 4:**
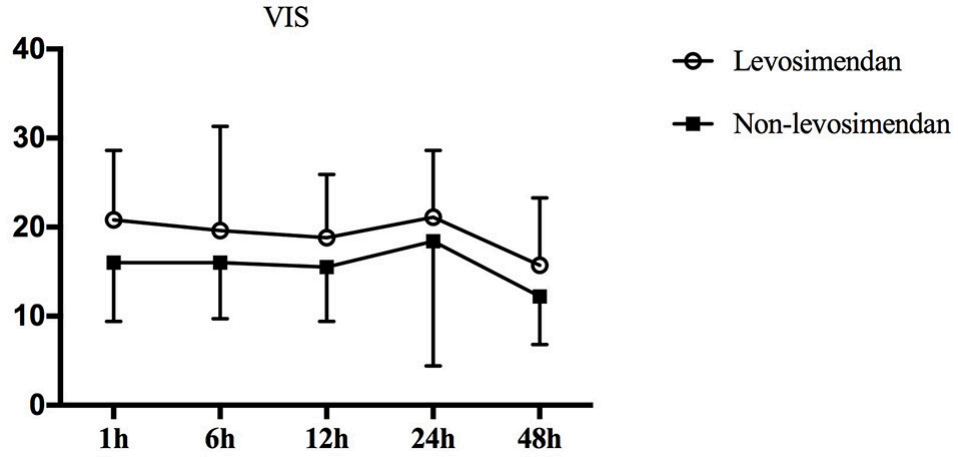
Repeated measures of analysis of VIS variation over time in the two groups. *p*_group._ = 0.093, *p*_time._ = 0.008, *p*_time × *group*._ = 0.853. Therefore, levosimendan had no influence on VIS after surgery. VIS, vasoactive-inotropic score.

All 40 pediatric patients were simultaneously supported by dopamine at the time of repair and in the ICU. It was found that during surgery, 95% (19/20) of the levosimendan group required epinephrine to maintain cardiac contractility compared with 65.0% (13/20) of the non-levosimendan group (*p* = 0.044). After admission to the ICU, four patients (20%) in the levosimendan group were maintained with vasopressin; however, no patient in the non-levosimendan group required vasopressin (*p* = 0.106; Table 2). Moreover, repeated measures analysis of variance for VIS implied no significant difference in the two groups (*p* = 0.093), nor was there any significant difference of interaction between group and time point (*p* = 0.853). There were significant changes in the postoperative VIS across different the time points of 1, 6, 12, 24, and 48 h (*p* = 0.008; Figure 4).

**Table 2:** Requirement for additional circular support regimens during perioperation.

	**Levosimendan (*n* = 20)**	**Non-levosimendan (*n* = 20)**	***p*-value**
**INTRAOPERATIVE MEDICAL CIRCULAR SUPPORT**			
^*^ Dopamine, *n* (%)	All	All	—
^*^ Dobutamine, *n* (%)	18 (90)	15 (75)	0.407
^*^ Epinephrine, *n* (%)	19 (95)	13 (65)	0.044
^*^ Norepinephrine, *n* (%)	4 (20)	2 (10)	0.661
^*^ Milrinone, *n* (%)	14 (70)	14 (70)	1.000
^*^ Vasopressin, *n* (%)	Null	Null	—
**POSTOPERATIVE MEDICAL CIRCULAR SUPPORT**			
^*^ Dopamine, *n* (%)	All	All	—
^*^ Dobutamine, *n* (%)	20 (100)	17 (85)	0.231
^*^ Epinephrine, *n* (%)	20 (100)	17 (85)	0.231
^*^ Norepinephrine, *n* (%)	2 (10)	2 (10)	1.000
^*^ Milrinone, *n* (%)	18 (90)	18 (90)	1.000
^*^ Vasopressin, *n* (%)	4 (20)	0 (0)	0.106

### Other postoperative outcomes

There was a significant tendency toward longer ICU stays for those in the levosimendan group than for those in the non-levosimendan group (median 10.5 days, IQR 7.3–39.3 days vs. median 4.0 days, IQR 2.0–10.0 days, *p* = 0.002). All patients received continuous mechanical ventilation after being transferred to the ICU except for one patient who was extubated in the operating room for fast-track anesthesia, thus we did not include the duration of that patient's mechanical ventilation. The comparison of mechanical ventilation between groups did demonstrate statistical significance (median 146.0 h, IQR 76.5–888.0 h for the levosimedan group; median 27.0 h, IQR 11.0–75.0 h for the non-levosimendan group, *p* = 0.002). There were eight patients who underwent continuous or intermittent peritoneal dialysis in the levosimendan group whereas there were only two in the non-levosimendan group (*p* = 0.028). The incidence of AKI was 75% (15/20) in the levosimendan group and 95% (19/20) in the non-levosimendan group, which was not significant (*p* = 0.184).

In the non-levosimendan group, three patients had to be supported by extracorporeal membrane oxygenation (ECMO) because of difficulty in weaning off CPB due to LCOS (their preoperative LVEF was 29.2, 25, and 40%); ECMO support lasted for 4, 7, and 7 days, respectively. Eventually, all patients were successfully separated from CPB. Throughout the 180 postoperative days to follow-up, all-cause mortality was 10% (2 of 20) in the levosimendan group compared with 5% (1 of 20) in the non-levosimendan group, and three deaths occurred in the ICU. In the levosimendan group, two deaths were attributed to left ventricular failure on postoperative days 42 and day 40 (LVEF values at baseline were 10 and 34%, respectively), and the one death in the non-levosimendan group was attributed to extreme LCOS accompanied by acute respiratory distress syndrome on day 17 (LVEF value at baseline was 20%; Table 3).

**Table 3:** The outcomes after left coronary artery reimplantation.

**Postoperative outcomes**	**Levosimendan (*n* = 20)**	**Non-levosimendan (*n* = 20)**	***p-*value**
ICU duration, days, median (IQR)	10.5 (7.3–39.3)	4.0 (2.0–10.0)	0.002
Mechanical ventilation, hours, median (IQR)	146.0 (76.5–888.0)	27.0 (11.0–75.0)	0.002
AKI, *n* (%)	15 (75)	19 (95)	0.184
Risk	9 (45)	11 (55)	
Injury	5 (25)	8 (40)	
Failure	1 (5)	0 (0)	
Peritoneal dialysis, *n* (%)	8 (40)	2 (10)	0.028
All-cause mortality, *n* (%)	2 (10)	1 (5)	1.000
Arrhythmia, *n* (%)	3 (15)	2 (10)	1.000
ECMO, *n* (%)	0 (0)	3 (15)	0.231
Re-intubation, *n* (%)	6 (30)	2 (10)	0.235
Tracheotomy, *n* (%)	4 (20)	0 (0)	0.106
Postoperative pneumonia, *n* (%)	5 (25)	6 (30)	0.723

*ICU intensive care unit, AKI acute kidney injury; ECMO, extracorporeal membrane oxygenation. In the item of mechanical ventilation, the sample size in levosimendan group was 19 for one patient was conducted for fast track and extubated in the operating room*.

## Discussion

In our study cohort, levosimendan seemed to have a favorable association with post-surgical cardiac function recovery because the improvement of ΔLVEF was twice that of the non-levosimendan group on day 7, although no statistical significance was found and no trend of increasing VIS score was manifested in the levosimendan group. Furthermore, in the levosimendan group, no patient required ECMO support after infusion of levosimendan, whereas three patients in the non-levosimendan group did require ECMO because of poor cardiac conditions.

To the best of our knowledge, this is the first analysis that explores the effect of levosimendan solely within a pediatric population with ALCAPA with mildly or severely impaired left ventricular function undergoing left coronary artery reimplantation, although it is a retrospective study. In fact, there are very limited studies regarding levosimendan in the field of pediatric cardiac surgery compared with the adult population. Moreover, all of these studies were conducted in the field of pediatric congenital cardiac repair rather than zoning in on a unique complex lesion. The occurrence of ALCAPA itself carries a relatively higher risk, and the surgical reimplantation is defined as the risk-adjusted classification for congenital heart surgery category 3; and it is also characterized by critically poor cardiac function prior to repair.

In terms of the timing of levosimendan administration, there have been some different practice preferences revealed in clinical pediatric settings. Anesthesiologists in our center have adopted the mindset that levosimendan should be administered at the discretion of the clinician as early as possible, and generally be started at the induction of anesthesia to decrease the incidence of postoperative LCOS and to prevent any unwanted consequences. The retrospective but single-group analysis conducted by Amiet et al. (15) involving 62 pediatric patients in the ICU after cardiac surgery demonstrated that using levosimendan as a rescue treatment once LCOS occurred could increase central venous oxygen saturation and reduce lactate 24 h later. They reported that in their clinical routine, they even infused the drug 24 h before various complicated surgeries in newborns. On other occasions, levosimendan was only regarded as a Supplementary agent during CPB weaning (16–18) or even as a rescue when difficulty arose (15). The LEVO-CTS (10) trial even claimed that levosimendan administration started just before surgery was actually not effective enough to reduce or avoid cardiac damage.

Clinical work with levosimendan has supported the drug's efficacy in improving cardiac function, as shown by the majority of trials in the pediatric population, but the meta-analysis conducted by Hummel et al. (19) involving five randomized controlled trials where all 212 patients were younger than 5 years old and undergoing congenital heart surgery summarized that when compared with standard treatments, prophylactic levosimendan in fact had no clear beneficial effect on LCOS. Momeni et al. (20) adopted heart rate × systolic blood pressure as an indicator of cardiac oxygen demand among neonates and infants undergoing congenital cardiac surgery; compared with milrinone, levosimendan was demonstrated to decrease this rate-pressure index at 24 and 48 h postoperatively. Ricci et al. (16) demonstrated that after a 72-h infusion, levosimendan appeared to be an excellent inodilator and more potential to improve the postoperative hemodynamic state persistently than the standard inotropic regimens among neonates with risk-adjusted classification for congenital heart surgery categories three and four and with the use of CPB. Therefore, it is suggested that levosimendan is the best and most commonly used drug for occasions where other routine inotropic therapies are not adequate to maintain hemodynamic stability. This is particularly the case for seriously impaired left ventricular function (21, 22). Just as in our practice, levosimendan was appropriately administered to patients with ALCAPA who had initial lower LVEF. In theory, levosimendan improved cardiac output, and its vasodilation trait can effectively decrease systematic vascular resistance and avoid pressure or volume overload in the management of hemodynamics during ALCAPA surgical repair. In such cases, after the left coronary artery is re-implanted, levosimendan's coronary-dilating property could be a great asset to assure adequate oxygen supply for injured myocardial tissue. In addition, due to the pharmacodynamic properties of levosimendan, a 24-h infusion can prolong its hemodynamic effects for ~7 days (23). Thus, this drug can support children through the most difficult postoperative phase in the ICU—that of postoperative cardiac deterioration. Moreover, ΔLVEF, reflecting the improvement of cardiac function to a more precise degree, was also greatly increased in the levosimendan group on day 7, Albeit cardiac functions were worse prior to surgical intervention. Our findings were consistent with the prospective clinical trial conducted by Lechner et al., who enrolled 39 neonates and infants undergoing open heart surgery and then compared the effect of prophylactic levosimendan against prophylactic milrinone administered after CPB. This trial indicated that changes in cardiac index were similar despite the fact that the levosimendan cohort presented with lower cardiac output prior to cardiac repair (17). Optimistically, there was no increase in VIS within 48 h and no ECMO requirement presented after levosimendan was used, which also indirectly affirmed its role in cardiac protection after a period of worse cardiac function prior to surgery. However, throughout the whole perioperation period, the number of patients requiring catecholamine administration in the levosimendan group was slightly higher than that of the non-levosimendan group; in particular, the use of epinephrine reached statistical significance. In the long-term follow-up to day 180, the ΔLVEF in the levosimendan cohort could be improved although it was not significant (*p* = 0.064 between the two groups); however, this is likely to be caused by multiple factors because it cannot be attributed to the performance of levosimendan independently.

Pediatric patients given levosimendan in our cohort had a longer ICU length of stay and mechanical ventilation support. These two unsatisfactory events may be associated with their persistent myocardial dysfunction. This may have also been a result of more patients requiring a second intubation for respiratory insufficiency and even necessitated a tracheotomy due to longer ventilation or deteriorating pneumonia. However, the requirement for peritoneal dialysis was just 4-fold that of the non-levosimendan group (8/2). In Ricci's cohort, renal function was replaced by urine output and peritoneal dialysis use, and a neutral outcome after levosimendan administration was demonstrated (16). However, a retrospective series conducted by Amiet et al. found that levosimendan could increase diuresis from 1.1 to 3.5 mL/kg/h by improving cardiac output (15). In our clinical practice, all 10 cases of peritoneal dialysis were applied to correct a series of consequences such as oliguria or anuria, which was secondary to the postoperative cardiac function of critically ill patients. Therefore, levosimendan is likely to have no potential in helping to avoid cardiac-renal syndrome among children with ALCAPA undergoing repair, but the incidence of AKI was 20% lower in the levosimendan group than in the non-levosimendan group. In our study, AKI was defined as early AKI that occurred within 12 h of the operation, and some late AKI cases were not identified because of the lack of late serum creatinine levels.

In fact, it is not certain that levosimendan, in our study, had any negative effect on postoperative 180-days all-cause mortality in our limited pediatric population despite the fact that two deaths occurred in the levosimendan group and only one in the control group. At present, only a few trials have discussed mortality in pediatric cohorts and negative conclusions have prevailed. In Ricci's study (16), there was also concern about mortality in the ICU among neonates undergoing cardiac surgery, with no reduced mortality observed in the levosimendan group. The meta-analysis mentioned previously (19) summarized that prophylactic levosimendan had no positive influence on mortality. Furthermore, the two multi-center and placebo-controlled trials published recently, the CHEETAH trial (11) and LEVO-CTS trial (10), have demonstrated that levosimendan could not reduce postoperative 30- and 90-days mortality, respectively, among adult patients with left ventricular dysfunction requiring cardiac surgery.

At present, levosimendan has been reported in most of the literature on regarding pediatric patients as having no adverse effects and as being well-tolerated. For instance, the randomized controlled trials of both Momeni (20) and Ricci (16) indicated that the postoperative heart rate in the levosimendan group was significantly lower than that of their own control groups. In a retrospective observational study, only one of 32 pediatric patients receiving levosimendan developed severe hypotension, with a diastolic blood pressure below 45 mm Hg, and the infusion had to be stopped 5 h later (24). However, we did record that after infusing levosimendan, there was one ventricular tachycardia case, one case of ventricular premature beat, and one supraventricular tachycardia case; however, no case of atrial fibrillation was observed. In the non-levosimendan group, however, there was one patient with atrial fibrillation and another with ventricular fibrillation. All of these cases were immediately treated with lidocaine or amiodarone. After a 24-h infusion, four patients in the levosimendan group required vasopressin to correct extreme hypotension because two cases could not be corrected with a massive dosage of norepinephrine, and another two had to be directly supported with vasopressin due to the patient's critical condition. We speculated that this was most likely due to levosimendan because of its vasodilation effect, although no loading dosage was given and the infusion speed was within a safe range. No administration of levosimendan was stopped during the therapy process. Whether hypotension, tachycardia, or other arrhythmias were incurred because of levosimendan administration is not certain, because this series is a retrospective study and multiple uncertain variables coexist.

Our study cohort highlights the notion that the prophylactic infusion of levosimendan is an ideal inotrope for preventing the deterioration of cardiac function after surgical intervention for ALCAPA, although other postoperative outcomes and mortality must be further investigated by future prospective and large-sample trials among pediatric patients. Another notable future direction is that levosimendan is indicated for the critically ill population who suffer from marked cardiac dysfunction before surgery.

There are some limitations to our study that must be considered. First, it is a single-center retrospective, non-randomized study and some unknown heterogeneities that we were unable to control had some impact on our analysis. The phenomenon of ALCAPA itself is a malign cardiac anomaly, and our perioperative hemodynamic management tactic is one of the most pivotal steps to support these patients through this difficult treatment phase. However, patient prognosis is very dependent on early diagnosis and early surgery and is also associated with individual preoperative cardiac function and surgical technique. Therefore, it is not clear whether the lack of improvement in LVEF was totally attributable to levosimendan in our study. Second, the infusion rate differed among individuals although it was always within a safe range (0.1–0.2 μg/kg/min) because every anesthesiologist carried out his/her appropriate inotropic scheme based on the patient's preoperative condition. Third, no related continuous biomarker measurements were acquired in our study, thus we could not make an evaluation of metabolic and cardiac injury conditions as a result of levosimendan. Fourth, the lack of long-term outcomes such as mortality, readmission to hospital, and the occurrence of heart failure limit our study. Finally, only a small number of patients with ALCAPA were enrolled, as it is a rare congenital heart disease, and this could bias the result. Overall, the current available literature is not sufficient to point out the benefits and risks of levosimendan in the pediatric population, especially for extremely critical or complicated congenital cardiac surgery, and further academic work is required to address its clinical effectiveness.

## Conclusions

The prophylactic infusion of levosimendan is confirmed to be a beneficial therapy in favor of recovering cardiac function among pediatric patients with ALCAPA and impaired left ventricular function who undergo surgical repair. However, this is a retrospective and observational analysis, and more rigid prospective studies are required to investigate its effect on a series of postoperative outcomes in the future.

## Ethics statement

This retrospective study was approved by the Institutional Review Boards of Fuwai Hospital, and informed consent was waived because of its retrospective nature.

## Author contributions

All authors contributed extensively to the work presented in this paper. YW, JG, and CW proposed the idea of this investigation. SS, JW, YG, SW, and JS were responsible for the collection of data and material. CW helped with the statistical analysis and wrote the manuscript. YW and YP helped to revise the manuscript. All authors read and approved the final manuscript.

### Conflict of interest statement

The authors declare that the research was conducted in the absence of any commercial or financial relationships that could be construed as a potential conflict of interest.

## Abbreviations

ALCAPA: anomalous origin of the left coronary artery from the pulmonary artery
LVEF: left ventricular ejection fraction
LCOS: low cardiac output syndrome
CPB: cardiopulmonary bypass
ECMO: extracorporeal membrane oxygenation
ICU: intensive care unit
VIS: Vasoactive-Inotropic Score
AKI: acute kidney injury
pRIFLE: pediatric-modified risk, injury, failure and loss and end-stage.
